# A Hop-Count Analysis Scheme for Avoiding Wormhole Attacks in MANET

**DOI:** 10.3390/s90605022

**Published:** 2009-06-24

**Authors:** Shang-Ming Jen, Chi-Sung Laih, Wen-Chung Kuo

**Affiliations:** 1 Department of Electrical Engineering, National Cheng Kung University / EE Building 92975R, No.1, Da-Shuei Rd., Tainan City 701, Taiwan; E-Mails: smjen@crypto.ee.ncku.edu.tw (S.-M.J.); laihcs@eembox.ncku.edu.tw (C.-S.L.); 2 Department of Computer Science & Information Engineering, National Formosa University / No.64, Wunhua Rd., Huwei Township, Yunlin County 632, Taiwan

**Keywords:** *ad hoc* network, hop-count analysis, MHA, network security, wormhole attack

## Abstract

MANET, due to the nature of wireless transmission, has more security issues compared to wired environments. A specific type of attack, the *Wormhole attack* does not require exploiting any nodes in the network and can interfere with the route establishment process. Instead of detecting wormholes from the role of administrators as in previous methods, we implement a new protocol, MHA, using a *hop-count analysis* from the viewpoint of users without any special environment assumptions. We also discuss previous works which require the role of administrator and their reliance on impractical assumptions, thus showing the advantages of MHA.

## 1. Introduction

The mobile *ad-hoc* network, MANET [1], is a developing wireless technology that has been discussed in many academic research projects in the last decade. An *ad-hoc* network is inherently a self-organized network system without any infrastructure. Typically, the nodes act as both host and router at the same time, i.e., each node in the network can be independent and based on different hardware, but when communication is needed it serves as a data transmitting router after a route discovery procedure.

So far, many routing protocols have been proposed for MANET, such as DSDV (Destination Sequence Distance Vector) [2], DSR (Dynamic Source Routing) [3] and AODV (Ad-hoc On-Demand Vector) [4] and so on. To the best of our knowledge, most previous research has focused on protocol establishment and its efficiency in MANET, but secure routing is very important, and some secure routing protocols based on DSR and AODV [5-7] have been proposed in these years.

**Figure 1.**
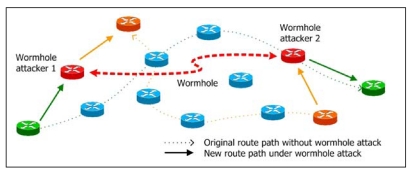
The wormhole attack in MANET.

Recently, a novel exploit called *wormhole attack* was introduced [8]. In a wormhole attack, attackers “tunnel” packets to another area of the network bypassing normal routes as shown in Figure 1. In practice, attackers can use high power antennas or a wired link, or other methods. The resulting route through the wormhole may have a better metric, i.e., a lower hop-count than normal routes. With this leverage, attackers using wormholes can easily manipulate the routing priority in MANET to perform eavesdropping, packet modification or perform a DoS (Denial of Service) attack, and so on. The entire routing system in MANET can even be brought down using the wormhole attack. Its severity and influence has been analyzed in [9].

Most previous works protecting against wormhole attack use methodologies assuming the viewpoint of administrator, trying to identify the wormhole, and then defend against it. They can further be classified as centralized systems like MDS-VOW (*Multi-Dimensional Scaling -Visualization of Wormhole*) [10], and distributed systems such as LBK (*Local Broadcast Key*) [11]. Some require substantial calculation and some others employ special nodes in the network. These methods consume effort and bandwidth as overhead. There are other similar schemes such as TIK protocol, SAM, DelPHI and LITEWORP which will be summarized in Section 2.

Instead of the viewpoint of administrator, we adopt the viewpoint of users and utilize routing information already available in standards like RFC3561. The concept is that we don't have to spend a lot of effort to catch thieves like the police (administrators), but rather lock the doors and windows as citizens (users), which is much easier and can avoid most of the threats with minimum effort. Our method selects routes and “avoids” rather than “identify” the wormhole resulting in low cost and overhead. We propose a multipath routing protocol called *Multipath Hop-count Analysis* (MHA, for short) to avoid wormhole attacks based on a *hop-count analysis* scheme. It is a highly efficient protocol which does not require any special supporting hardware. Furthermore, MHA is designed to use split multipath routes, so the transmitted data is naturally split into separate route. An attacker on a particular route can not completely intercept (and subvert) our content.

The rest of the paper is organized as follows: We review related works regarding wormhole attack in Section 2. In Section 3, the MHA protocol is proposed. The simulations are given in Section 4, and then comparison and discussion are provided in Section 5. Finally, we present our conclusions and future work in Section 6.

## 2. Related Works

In this section, we review related works in the literature which discuss proposed wormhole attack defenses.

### 2.1. Graph Theoretic Approach

Lazos *et al.* [11] proposed a graph theoretic model to characterize the wormhole attack and ascertain the necessary and sufficient conditions for any candidate solution to prevent wormholes. They used a *Local Broadcast Key* (LBK) based method to set up a secure *ad-hoc* network against wormhole attacks. In other words, there are two kinds of nodes in their network: guards and regular nodes. Guards access the location information through GPS or some other localization method like SeRLoc [12] and continuously broadcast location data. Regular nodes must calculate their location relative to the guards' beacons, thus they can distinguish abnormal transmission due to beacon retransmission by the wormhole attackers. All transmissions between node pairs have to be encrypted by the local broadcast key of the sending end and decrypted at the receiving end. As a result, the time delay accumulates per node traveled. In addition, special localization equipment has to be applied to guard nodes for detecting positions.

### 2.2. Packet Leashes

In [9], Hu *et al.* introduced a packet leashes method to restrict the time that packets can be transferred. They propose the TIK protocol based on *TESLA* [13] and use *temporal leashes* to determine the wormhole attack by transmission time. Consequently, TIK requires precisely synchronized time among the nodes. In addition, TIK combines hash tree authentication to ensure the time information in the control packet is not modified. Therefore, the receiver can confirm if the packet transmission distance satisfies the restriction that sender has claimed. The TIK packet is transmitted by *S* as:

$$S\to R:<{\text{HMAC}}_{K_{i}}\left(M\right),M,T,K_{i}>$$

Where:

HMAC*_K_i*(*M*): HMAC for verifying the content;
*M*: Plaintext;
*T*: Values needed for authentications;
*K_i_*: The key for time interval *T_i-1_*∼*T_i_*.

Before the sender sends a packet *P*, it estimates an upper bound *t_r_* on the arrival time of the HMAC at the receiver. Then it picks a key *K_i_* which will not expire before the receiver gets the packet's HMAC, i.e., *T_i_* > *t_r_* + Δ, where Δ is the synchronized error between sender and receiver. Next, the sender computes the HMAC*_K_i*(*M*) with *K_i_* and attaches the HMAC to the packet. The sender then discloses the key *K_i_* after the key has expired. After the receiver obtains the HMAC, it first checks if the key is expired. If the sender has not sent the corresponding key *K_i_*, the key is available. The receiver later uses the hash tree root *m* and the hash tree value *T* to verify the *K_i_* at the end of the packet authentication, then it uses the authenticated *K_i_* to verify the HMAC value in the packet. If all these verifications are correct, the packet is accepted as authenticated.

However, the assumptions of TIK are impractical. It depends on precisely synchronized time between all nodes and assumes the packet sending and receiving delays are negligible. The wormhole is discovered because it passes packets more slowly than normal routes. Furthermore, knowledge of the positions of all nodes may be a prerequisite for correctly estimating transmission times.

### 2.3. Other Protocols and Mechanisms

In [10], Wang *et al.* designed MDS-VOW, a topology visualization system, to visualize the network topology of a sensor network and detect wormholes. In [14], Qian *et al.* presented the SAM protocol which analyzes the frequency of nodes used, and flags overuse as abnormal. In [15], Maheshwari *et al.* used the information of connectivity to find wormhole. In [16], Naït-Abdesselam *et al.* used link information for wormhole detection based on the OLSR protocol. There are other works that focus on defending against wormhole attacks in MANET, such as DelPHI in [17] and LITEWORP in [18], and so on. However, most of these mechanisms require some special assumptions and supporting hardware, and some of them are based on specific protocols.

## 3. A Proposed Robust Scheme Based on Hop-Count Analysis (MHA)

### 3.1. The Concept of MHA

**Figure 2.**
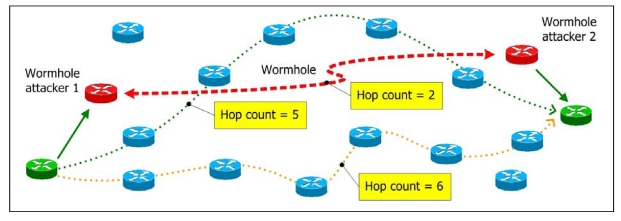
Distinguishing safe routes from hop-count values.

We illustrate the concept of MHA in Figure 2. The normal routes for a particular communication pair contain 5∼6 hops; the route under a wormhole attack has a hop-count of 2. We see the route under the wormhole attack has a smaller hop-count than normal. As a result, users who avoid routes with relatively small hop-counts can avoid most wormhole attacks.

In MHA, we first examine the hop-count values of all routes. Then we choose a safe set of routes for data transmission. Finally, we randomly transmit packets through safe routes. Even if the wormhole is not avoided in some severe cases, we can still minimize the rate of using the route path through the wormhole. The simulation results are shown in Section IV.

**Figure 3.**
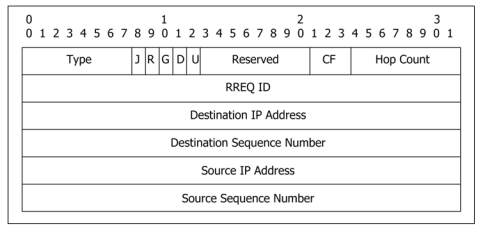
RREQ packet format of MHA protocol.

In our work, we refer to RFC3561 [4], i.e., the AODV routing protocol, to set up MHA. We use the control packets as in RFC3561 and modify them to satisfy our requirements. Their modified functions are defined in the following section. For example, the RREQ packet is used for route discovery and the RREP packet is used for route reply, and so on. Figure 3 shows the RREQ packet format in MHA. While most fields stay as they were in RFC 3561, in addition, we define a field called *check flag* (CF), which is used with RREQ ID to distinguish new RREQ packets from old ones in MHA. The CF is also included in the RREP packet. A RREQ packet with CF = 0 is served as a normal route discovery, while CF ≠ 0 represents the *graylist broadcast*, which is introduced in section 3.3. The source node triggers the graylist broadcast based on the result of hop-count analysis.

The MHA contains four parts. The route establishment is introduced in Section 3.2, and graylist broadcast is illustrated in 3.3. The hop-count analysis scheme is proposed in 3.4. Finally, the route maintenance method is given in 3.5. The notations used are listed in Table 1.

### 3.2. Route Establishment in MHA

**Figure 4.**
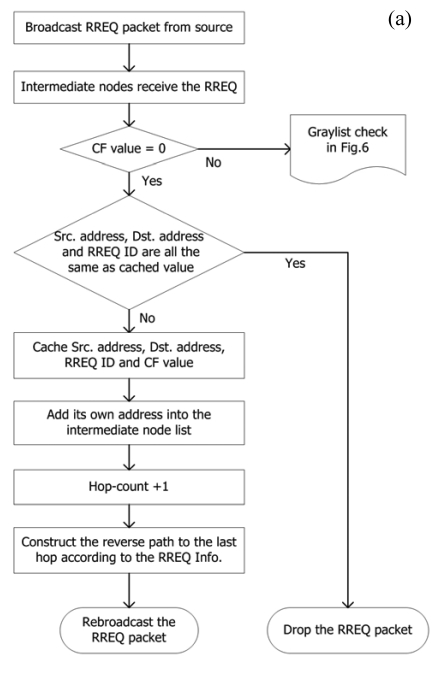

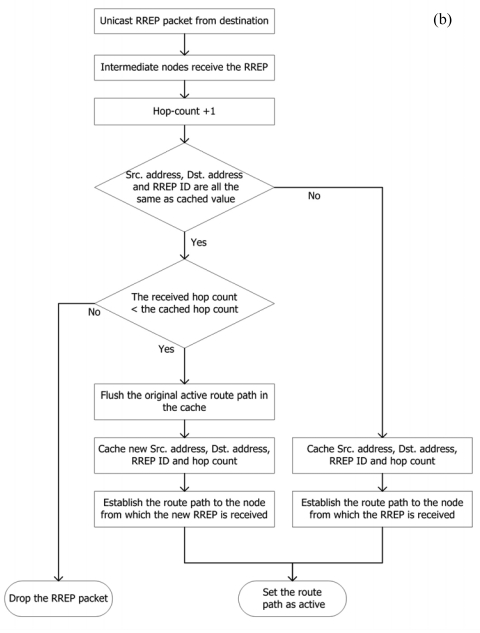
Route establishment in MHA protocol. (a) Processing a RREQ packet. (b) Processing a RREP packet.

The source node launches a route discovery procedure if there is a communication requirement. It first adds IP_S_, IP_D_ and RREQ ID in the RREQ packet, then sets the hop-count = 0 and attaches an expiration time to the RREQ packet. Later, the RREQ packet is broadcasted into the network and received by surrounding nodes. In Figure 4(a), the nodes which receive the RREQ packet process it as follows:

1. Check CF to confirm the broadcast type. When CF = 0, examine IP_S_, IP_D_ and RREQ ID with cached values;
  - If all values are the same, drop the packet;
  - If not, cache IP_S_, IP_D_, RREQ ID and CF;
2. Add its own IP address into the intermediate list;
3. Hop-count + 1;
4. Construct a route back to where the RREQ packet came from;
5. Rebroadcast the RREQ packet and switch to waiting mode. The nodes in this mode can only used for transferring control packets.

With the flooding of RREQ packets, the destination node finally receives them. The destination node then generates the RREP packets with following steps:

1. Copies IP_S_, IP_D_, RREQ ID and CF from the RREQ packet and writes in the corresponding field of the RREP packet, then sets the hop-count = 0;
2. Unicasts the RREP packet to the source node through each route.

In Figure 4(b), when intermediate nodes receive the RREP packet, they process it as follows:

1. Hop-count + 1;
2. Compare IP_S_, IP_D_, RREP ID and CF with cached values;
  - If they are all the same, i.e., there already exists a route to the destination node, compare the new hop-count in the RREP packet with cached value. If the new one is smaller, or the lifetime of the original route expired, replace the original route with the new one and renew the corresponding fields with values in the new RREP packet; otherwise, drop it;
  - If any of them are different, cache IP_S_, IP_D_, RREP ID, CF and hop-count.

The destination node then establishes a route back to where the RREP packet came from and sets the route as active thus it can be used for transferring data packets.

### 3.3. RREP number limit and Graylist Broadcast

**Figure 5.**
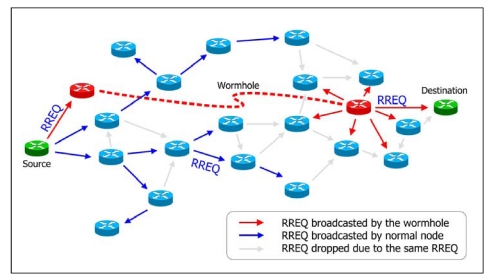
Route establishment under the wormhole attack.

The RREP packets are finally sent back to the source node. In our MHA protocol, we set a *RREP number limit* (RREP_lim_) to consider the reasonable amount of routes that are found in a route discovery procedure. This is designed for a special circumstance in attacking RFC 3561. In Figure 5, there is a wormhole between a communication pair. Due to the better metric of the wormhole, the RREQ packet rebroadcast by the wormhole is accepted by the nodes around the destination node at first. Consequently, the RREQ packets which are flooded from the normal nodes later will be dropped. As a result, only the route through the wormhole can be established.

The RREP_lim_ can prevent this situation. This value can be adjusted according to the network circumstances. For example, in a highly trusted environment, where most nodes consist of familiar people, we can safely decrease the RREP_lim_ value in the route discovery procedure. First, we set the default RREP_lim_, such as RREP_lim_ = 2. After the source node receives all RREP packets, it compares the number of received RREP packets γ with RREP_lim_. If γ < RREP_lim_, it probably means that there is a wormhole, or too few nodes in the network. The source node then launches the graylist broadcast in this situation.

The graylist broadcast contains the following steps:

1. CF + 1 in the RREQ packet;
2. Adds all intermediate nodes which are found in the last route discovery procedure into the graylist;
3. Broadcasts the RREQ packet with the graylist.

**Figure 6.**
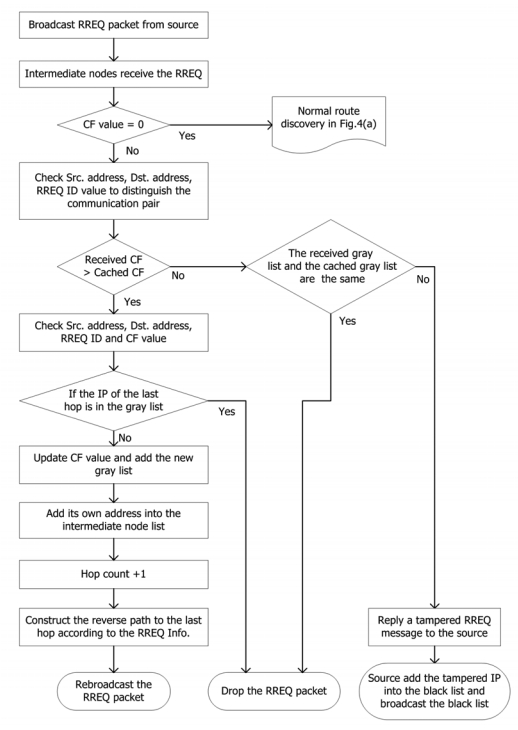
Graylist broadcast in MHA protocol.

In the graylist broadcast illustrated by Figure 6, the nodes handle the received RREQ packet in following steps:

1. Check CF to confirm the broadcast type. When CF ≠ 0, check IP_S_, IP_D_ and RREQ ID to determine the communication pair;
2. Compare CF with the cached value;
  - If it is the same as the cached one, check the received graylist with the cached one;
    - If they are the same, drop the packet;
    - If some values in the graylist are suspected to be modified by the malicious nodes, alert the source node of the modified value. The source node then marks the suspect IP address in the blacklist The blacklisted nodes will not be used for a period;
  - If CF is larger than the cached one, update the CF and graylist in the memory and check if the last hop in the RREQ packet is in the graylist;
  - If the last hop has been recorded in the graylist, drop the packet; if not, add its own IP address into the intermediate list;
3. Hop-count+1;
4. Construct a route back to the RREQ packet came from;
5. Rebroadcast the RREQ packet and enter the waiting mode of this route.

The destination node receives the RREQ packet and replies RREP packets as usual. Finally, the source node receives the new RREP packets with a larger CF. The source node compares γ with RREP_lim_ again and triggers another graylist broadcast until at least one of the following conditions is true:

1. γ ≥ RREP_lim_;
2. CF = 7. Considering efficiency, CF value in MHA reserves 3 bits, i.e., CF_max_ = 7;
3. No RREP packet reply, perhaps due to a too small quantity of nodes in the network.

After the source node completes the route discovery and graylist broadcast, it continues to the hop-count analysis and route path selection mechanism.

### 3.4. Hop-Count Analysis Scheme and Route Selection

In MHA, a route with too low or too high hop-count is considered unhealthy. A too low hop-count may imply a wormhole attack; while a too high hop-count may decelerate the transmission. Furthermore, a route with a high hop-count has a higher chance to break.

In MHA, we define random variable *X* which represents the hop-count values in the received RREP packets, and *U* = {*x_1_*, …, *x_i_*, …, *x_j_*, … } is the sample space of random variable *X*. So we have its cumulative distribution function *F_X_(x)*. We further define variables *α* and *β* which represent the lower bound and upper bound of the selected range on *F_X_(x)*, then *m* and *n* will be the corresponding hop-count boundaries. They are defined in Equation (1) as follows:

(1) $$\begin{array}{c} x_{i},x_{j}\in U,i,j,m,n\in N\\ 0<\alpha <\beta <1\\ m=\sup\left\{x_{i}|F_{X}\left(x_{i}\right)\leq \alpha \right\}\\ n=\inf\left\{x_{j}|F_{X}\left(x_{j}\right)\leq \beta \right\} \end{array}$$

Finally, we select the route paths from received RREP packets with hop-count x_r_ which satisfies Equation (2):

(2) $$\begin{array}{c} r\in N,\cdot x_{r}\in U\\ x_{i}\leq x_{r}\leq x_{j} \end{array}$$

as legal route paths in MHA protocol. The legal route paths are cached in the source node and given a TTL. Data packets are then transmitted randomly through these routes. If any of these legal route paths are broken, the source node deletes it from the cache. If all legal route paths are broken or vanished, and there is still a requirement for communication, the source node will carry out another route request procedure.

In addition, the source node unicasts route acknowledgement (RACK) packets along all legal routes to the destination node before the first data packet is sent. The RACK packet contains the intermediate nodes of all legal routes and the expiration time. The destination node then caches them for further transmission. Once this step is completed, the bidirectional routes of a communication pair are established.

### 3.5. Route Maintenance in MHA Routing Protocol

#### 3.5.1. Dealing with Broken Routes and RERR Packets

As time passes, routes may break. When a node receives a data packet but can not reach the next hop in the routing table, it generates a RERR packet including IP_S_ and IP_D_ to notify the source/destination node.

The source/destination node receives the RERR packet, deletes the broken route in memory and uses the RACK packet to notify the destination/source node to use other remaining routes. The destination/source node also deletes the route. Until the next route discovery procedure, the remaining routes are used for transmission.

#### 3.5.2. Cross-check the Route Paths

In the MHA protocol, we give an expiration time for every route. All intermediate nodes delete the cached route after expiration to conserve memory. However, in certain environments, the nodes in the network may be static. These routes may persist over time. In order to have better efficiency, when routes of a communication pair are expired but are still in use, the source node unicasts a RACK packet with a new expiration time to the destination node through the valid routes. The intermediate nodes and the destination node then refresh the expiration time in their routing table.

Consequently, we limit resource usage and recover some overhead that would originally be wasted by launching unneeded route discovery procedures. We also save memory of intermediate nodes that would otherwise be used for keeping idle routes.

## 4. Simulations

To test our method, we develop an event driven simulator by using C and Matlab. The C program is used to set up the simulation environment and compute the actions of all nodes between route discovery processes. Then, we visualize the simulation results by using Matlab.

We assumed each node is distributed randomly by the simulator, and each node will send and receive packets with different delays in an allocated range. The source establishes a route discovery and graylist broadcast by sending the RREQ packet. In response, the destination node replies with RREP packets. The source node then selects the legal routes.

Furthermore, the simulator allows users to input the RREP_lim_ value for each simulation. We can input different RREP_lim_ for each simulation, and the simulator completes the graylist broadcast as required.

In this section, we give four experiments to show the performance of the proposed protocol. In Table 2, we give the parameters used in the experiments.

### 4.1. Experiment 1 - Maximum Wormhole Effect

**Figure 7.**
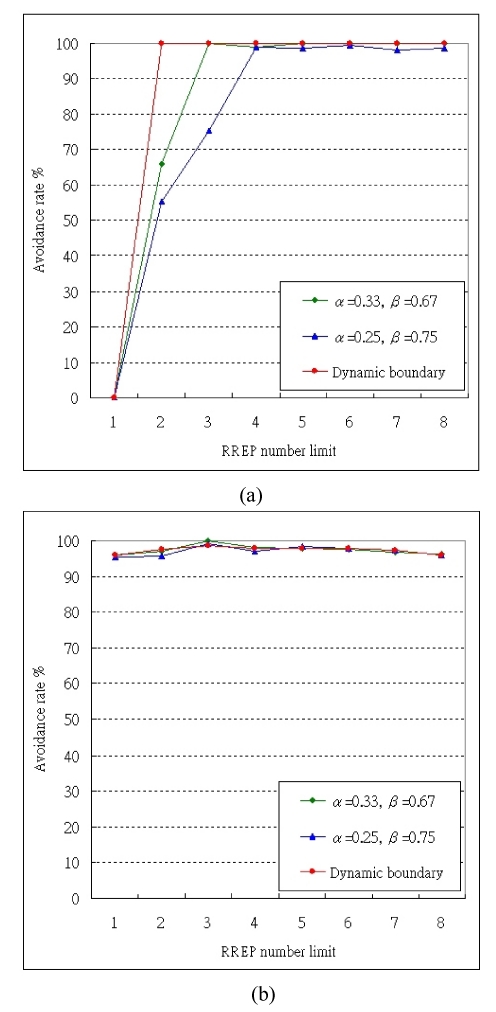
The comparison of avoidance rates in (a) Exp. 1 (b) Exp. 2.

In Exp. 1, we verify the avoidance rate of the maximum wormhole effect. That is, the wormhole is placed in the middle of the communication pair and other normal nodes are placed at random. Figure 7(a) shows the avoidance rate with different legal boundaries (α, β).

We apply three kinds of boundaries in this experiment. With loose boundaries of (0.25, 0.75), only the routes with hop-counts in the middle 1/2 distribution are selected as legal. As a result, the avoidance rate is higher than 98% when RREP_lim_ ≥ 4. With stricter boundaries of (0.33, 0.67), the avoidance rate is higher than 99% when RREP_lim_ ≥ 3.

Nevertheless, we design a dynamic boundary. We apply different boundaries (α, β) according to the received RREP numbers, e.g., we choose (0.5, 1) for received RREP number = 2, and (0.35, 1) for received RREP number = 3∼4, and (0.25, 0.75) for received RREP number = 5∼6, and so on. The dynamic boundary gives a better percentage in avoiding wormhole attacks and also enhances the performance by decreasing transmission distance in hops. Refer to Figure 5(a), as long as RREP_lim_ ≥ 2, the avoidance rate can reach 100%.

However, the wormhole attack is unavoidable when RREP_lim_ = 1 for any boundaries, because the only established route goes through the wormhole. As the RREP_lim_ increases, the avoidance rates of all boundaries raise. The effect on a strict boundary is more obvious than it is on a loose boundary.

### 4.2. Experiment 2 - Random Wormhole Effect

In Exp. 2, we verify the avoidance rate of the random wormhole effect, that is, all nodes including the wormhole and the communication pair are distributed at random. Then, we compare the effects of different boundaries and show its result in Figure 7(b).

In this experiment, the effect from adjusting boundaries is not as clear as it is in Exp. 1. This is because the random wormhole does not have the best metric to some particular transmission pairs in routing. In this case, the inherent multipath transmission nature of MHA exhibits protection against wormholes by splitting data into different routes, so the attacker cannot launch further attacks.

### 4.3. Experiment 3 & 4 - Overhead of MHA

**Figure 8.**
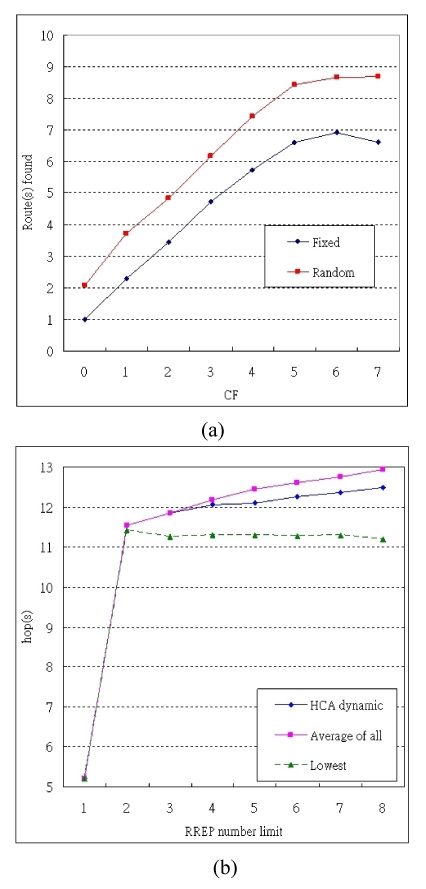
The overhead of MHA in (a) Exp. 3 (b) Exp. 4.

In Exp. 3, we examine the number of routes that have been found in each graylist broadcast under both maximum and random wormhole effect. The number of routes found for CF from 0 to 7 is shown in Figure 8(a).

In Exp. 4, we examine the transmission distance in MHA with dynamic boundary under the maximum wormhole effect. The lowest hops, i.e. the route found with AODV without wormhole, and average hops, i.e. the average of all available routes, are also shown together in Figure 8(b).

## 5. Discussion and Comparison

### 5.1. Discussion

In Exp. 1, the avoidance rate of maximum wormhole effect is verified. We find when MHA applied with constant boundaries, the strict boundaries performs better than the loose ones in avoiding wormhole attack and the simulation result is shown in the Figure 5(a). The curve of the strict boundary rises faster than the loose one with the growth of RREP_lim_ value. Furthermore, we present the dynamic boundary, which changes with the received RREP number, in order to improve accuracy and efficiency. As a result, it can avoid 100% of wormhole attacks when RREPlim ≥ 2. In Exp. 2, the avoidance rates for all RREP_lim_ are over 96%.

In Exp. 3, at CF = 1, i.e., only one graylist broadcast is needed, 2.3 and 3.74 routes are found on average under the maximum and random wormhole effects, respectively. Compared with Exp. 1 and Exp. 2, it performs with 100% and 96% avoidance rates, respectively. As a result, we can conclude that only one graylist broadcast is required for avoiding wormhole attacks, and further minimize overhead. In addition, multi-wormhole cases that have not been discussed in other previous works can be avoided by increasing the RREP_lim_ value in MHA. On the other hand, if there is no wormhole within a closed environment, the graylist broadcast can be cancelled by setting RREP_lim_ = 1 to save energy and bandwidth.

In Exp. 3, we also find in our assumed environment, most independent routes can be found when CF < 6. In Exp. 4, the transmission distance of MHA is longer than the shortest route by about one hop, but below the average of all routes. This is because with MHA, the routes with higher hops are also filtered out. In addition, the shortest route can represent the route discovered by AODV in safe environments. It shows our judicious use of an extra hop (on average) to grant us a great amount of protection against wormhole attacks in comparison to AODV.

Our method provides good performance for avoiding wormhole attacks, but there could be some attacks anticipating MHA. For example, attackers may add fake nodes to an intermediate list so the route has a longer distance. However, it is quite hard for attackers to correctly estimate the expected hops of a particular communication pair since they do not know their relative position. In addition, since we only select part of the searched routes for multi-path transmission, the probability that attacks can occupy the route are further reduced. In another scenario, attackers may maliciously modify other nodes instead of itself in the graylist. Thus the nodes that have been modified would be reported as modifiers and be blocked by the source node. To counter this, some ID-based cryptographic methods [19] such as digital signatures can be adopted to prevent this.

### 5.2. Comparison

Most of the protocols proposed in literature can reach high avoidance rates in their experiments. However, they all need some impractical assumptions or special hardware as listed in Table 3. The MHA routing protocol does not need impractical assumptions, such as precise synchronized time, zero delay time, or the awareness of all node locations, and so on, which are usually assumed in the schemes adopting the viewpoint of administrator. In addition, MHA does not need any special hardware either. Due to its simple concept, the hop-count analysis scheme can improve most routing protocols in MANET as well. The nodes only need to sort the received hop-counts, and select the legal ones according to the preset boundaries. Thus the computation overhead is very low for this process.

We compare MHA with previous works in Table 3. In [9], a large amount of hash tree computation is required. In their discussion, the optimized condition performs 10 million hash function evaluations in 7.544 seconds on a Pentium III running at 1 GHz; and 45 seconds on a Compaq iPAQ 3870 PocketPC running Linux. A long waiting time for system setup shows their high overhead. In [11], all transmissions between node pairs have to be encrypted by the local broadcast key of the sending end and decrypted at the receiving end. As a result, the time delay accumulates per node traveled. The MDS-VOW mechanism in [10] uses a control center to accomplish the *O(n^3^)* operations if there are n nodes. This is a fairly heavy load for the control center.

## 6. Conclusion and Future Works

In this paper, a novel scheme based on an intuitive method to avoid wormhole attacks in MANET is proposed. The defining characteristic of this method is avoiding wormhole attacks from the viewpoint of users instead of the administrator's viewpoint as in previous works. We provide four simulations to show the proposed scheme has high efficiency and very good performance with low overhead. In addition, this scheme does not require additional hardware or impractical assumptions of the networks. Hence, it can be directly used in MANET.

Although we have proposed a solution for the wormhole attack problem in MANET, the dynamic information of the packets could still be modified. This issue can be solved by some cryptographic method, such as a homomorphic one-way hash function. The static data, i.e. payload, can be secured by ID-based signature. These methods can also be adopted for MHA to give a more robust protection in some special scenarios like battlefields, which need a highly secured environment.

## Figures and Tables

**Table 1.** Notations.

**Parameter**	**Represents**
CF	The check flag value
IP_S_/IP_D_	The source/destination IP address
RREP_lim_	RREP number limit
γ	Number of received RREP packets
α, β	The lower, upper bound for route selection
m, n	The lowest, highest hop-count of legal routes

**Table 2.** Parameters for experiments.

**Parameter**	**Value**
Number of nodes	300
Field dimensions	2,000 m × 2,000 m
Radio range	250m
Node delay	Random @ 0.05∼0.075 ms
Trial	50 times

**Table 3.** Comparisons on related works and MHA.

**Mechanism**	**Special assumptions**	**Special hardware**	**Overhead**
Our proposed scheme (MHA)	No	No	Low

TIK-scheme [9]	Zero delay time on nodes	No	High
	Slower wormhole		
	Precisely synchronized time		
	Predictable transmission time		
	Off-line hash computation		

LBK-scheme [11]	Beacon retransmission of wormhole	Guards	High

MDS-VOW-scheme[10]	Sensor network	Control center	High
	Statistic topology		
	Constant radio strength		
